# Tracking SARS-CoV-2 genomic variants in wastewater sequencing data with *LolliPop*

**DOI:** 10.1371/journal.pcbi.1014003

**Published:** 2026-02-19

**Authors:** David Dreifuss, Ivan Topolsky, Pelin Icer Baykal, Niko Beerenwinkel

**Affiliations:** 1 Department of Biosystems Science and Engineering, ETH Zurich, Basel, Switzerland; 2 SIB Swiss Institute of Bioinformatics, Lausanne, Switzerland; Fundação Getúlio Vargas: Fundacao Getulio Vargas, BRAZIL

## Abstract

During the COVID-19 pandemic, wastewater-based epidemiology has progressively taken a central role as a pathogen surveillance tool. Tracking viral loads and variant outbreaks in sewage offers advantages over clinical surveillance methods by providing estimates not biased by testing practices and enabling early detection. However, wastewater-based epidemiology poses new computational research questions that need to be solved in order for this approach to be implemented broadly and successfully. Here, we address the variant deconvolution problem, where we aim to estimate the relative abundances of genomic variants from next-generation sequencing data of a mixed wastewater sample. We introduce *LolliPop,* a computational method to solve the variant deconvolution problem. *LolliPop* is tailored to wastewater time series sequencing data and applies temporal regularization in the form of a fused ridge penalty. We show that this regularization is equivalent to kernel smoothing and that it makes abundance estimates robust to very high levels of missing data, which is common for wastewater sequencing. We use the bootstrap to produce confidence intervals, and develop analytical standard errors that can produce similar confidence intervals at a fraction of the computational cost. We demonstrate the application of our method to data from the Swiss wastewater surveillance efforts as well as on simulated data.

## Author summary

Wastewater-based epidemiology has become a valuable tool for tracking viruses like SARS-CoV-2 across entire communities. Sequencing wastewater can reveal which viral variants are circulating, offering early insights into variant dynamics while avoiding the biases of clinical testing. A central challenge is to infer the relative abundances of these variants from observed mutation data. This task is complicated by the fact that variant profiles can be highly similar, and the data is often noisy with many missing read count values from genomic positions with no coverage, especially when the incidence of the pathogen is low. We developed *LolliPop*, a statistical method that leverages the time series structure of wastewater data to robustly deconvolve variant abundances and compute fast confidence intervals. Using both simulated data and real data from the Swiss national variant monitoring, we show that *LolliPop* is accurate and robust to high levels of missing data.

## Introduction

During the COVID-19 pandemic, genomic surveillance has been applied at an unprecedented scale to support various national efforts in containing outbreaks [1]. In this context, wastewater monitoring has seen its broadest and most successful application, with PCR-based surveillance of total viral loads sometimes complemented with next-generation sequencing (NGS) [2]. As genomic analysis is extended from clinical samples to samples from wastewater treatment plants (WWTPs), new statistical and computational research questions arise: the samples are mixtures of heterogeneous, possibly highly diverse RNA molecules, for which the traditional approach of reconstructing consensus sequences is not suitable [1]. Beyond SARS-CoV-2, wastewater-based epidemiology (WBE) is becoming a central tool for pathogen surveillance in general [2]. WBE has been shown to inform on the infection dynamics of pathogens for which clinical testing is usually low, such as influenza and respiratory syncytial virus [3,4]. It is therefore pressing that the computational challenges of analyzing mixed, heterogeneous wastewater sequencing data be addressed.

Most of the existing viral genomic data analysis pipelines and tools were designed for clinical samples and rely on classifying the majority variant of each sample from the consensus sequence of the read alignment [5–7]. One of the main challenges in analyzing wastewater-derived NGS data is the loss of information about the full viral haplotype, i.e., which mutations occur together on the same RNA molecule. This loss can result from fragmentation of the genetic material and from sequencing protocols, which often rely on PCR amplification of the target genome in multiple amplicons. In addition, the sequencing data exhibits very high levels of noise, because from a large pool of raw wastewater, extreme downsampling steps (grab or composite sampling, filtering, random reverse transcription, etc.) are followed by extreme amplification steps (PCR). This in turn can result in low genomic coverage and high levels of missing data. Some tools have been developed to increase sensitivity in the detection of variants, for example, by searching for co-occurring mutations on the same read, which has been shown to improve early detection of newly introduced variants [8].

Beyond early detection of new introductions, quantitative estimation of the relative abundances of different variants from wastewater is a very important aspect of viral genomic surveillance. The relative growth dynamics of a new variant can inform on its fitness advantage relative to the dominating strain [9], and hence on its predicted impact on the infection dynamics. The fitness advantage is an epidemiologically important parameter that can be estimated accurately from wastewater samples using either specific PCR-based assays [10,11] or NGS data [8,12,13], while using far fewer samples as compared to using clinical data, provided that accurate quantification of the variant relative abundances can be made. For practical planning and policy making, it is therefore crucial to have accurate and time-efficient methods for estimation of the relative abundances through time of variants from wastewater NGS data, including reliable measures of uncertainty.

Prior research has shown that the relative abundance of an emerging variant can be quantified by averaging over a set of mutations that is unique for this variant [8]. However, as the number of variants grows, shared mutations cannot generally be discarded as finding sets of unique, characteristic mutations for each variant quickly becomes inefficient or impossible. To address this limitation, some methods have been developed that take into account the correlation structure of mutations between different variants [14–17]. Some of these methods – such as *Freyja* [16] *–* also rely on deconvolution of the mutation frequencies in a sample into the relative abundance of variants by inverting a linear model. However, with these methods, quantification of uncertainty is done on the basis of the computationally intensive bootstrap, which can be prohibitive when faced with large amounts of data or limited computational resources (as is often the case in real-world surveillance settings). It is also not guaranteed in general that these methods fare well with high levels of missing data, which are common in environmental sampling due to low concentrations and high PCR inhibition [18].

Here, we introduce a new method for estimating variant relative abundances from wastewater sequencing data, named *LolliPop*. Tailored to time-series data, our method estimates the time course of relative abundances of all variants in the mixed sample. We employ temporal regularization to make this approach robust to high levels of missing data, which are frequent in wastewater sequencing. As an alternative to confidence bands based on the bootstrap, we derive analytical methods to compute asymptotic confidence bands, which provides a 30-fold speedup. We evaluate our method on simulated datasets with high missing-value rates, as well as on wastewater NGS data with low genomic coverage, where we find high correlation between the inferred variant abundances and estimates from matched clinical data [8]. *LolliPop* is currently used for the Swiss wastewater monitoring program (https://wise.ethz.ch/?page=viruses/variants).

## Methods

We model expected mutation frequencies in wastewater samples as a linear combination of variant profiles. To address challenges such as high noise, missing data, and similarity between variant profiles, we apply temporal regularization in the form of a fused ridge penalty. This leads to a regularized loss minimization framework for deconvolving relative variant abundances over time. We provide both bootstrap-based and analytical confidence intervals, incorporating adjustments for overdispersion and using logit reparametrization to ensure valid bounds and variance reduction. We evaluate the method on real wastewater sequencing data from the Swiss national surveillance program and on simulated datasets designed to assess robustness across varying levels of missing data, variant profiles similarity and model misspecification.

### Variant deconvolution

We consider an ordered collection of samples, taken at (not necessarily evenly spaced) timepoints t=1, ..., T . Each studied variant v∈{1,...,V} carries a subset of the mutations m∈{1,...,M} relative to a fixed reference strain. Let $X\in \{\text{0},\text{1}{\}}^{M\times \;V}$ be the design matrix of variant definitions, i.e., $X_{m,v}=\text{1}$ if variant v bears mutation m, and $X_{m,v}=\text{0}$ otherwise. Let $y_{t}\in [\text{0},\text{1}{]}^{M}$ be the observed mutation frequency vector at time t, where the entries are the observed proportions of reads from the wastewater sequencing experiment supporting a certain mutation m. We are interested in $b_{t}\in [\text{0},\text{1}{]}^{V},\;||b_{t}{\left|\right|}_{\text{1}}=\text{1},$ the relative variant abundances of all variants at each time point t. We make the assumption of a linear probability model, where at time t the probability of a read carrying a certain mutation equals the sum of the relative abundances of the variants bearing that mutation. We thus have that the expected proportion of reads with a given mutation is a linear combination of the variant relative abundances:


$${\;E[y}_{t}\;|{\;b}_{t}]=Xb_{t}$$ (1)

**Definition (Variant Deconvolution Problem):** For given variant definitions X  and a time series of mutation frequencies $y_{\text{1}},\;...,\;y_{T}$, the variant deconvolution problem is to find the relative variant abundances $b_{\text{1}},\;...,\;b_{T}$ in the population of the catchment area of the WWTP, such that ${\;y}_{t}=Xb_{t}$ for all time points *t*.

As finding the exact relative variant abundances in the population is not possible due to the randomness of the data-generating process and model misspecification, we relax the problem to finding a best approximation. Solving the variant deconvolution problem is then performed by choosing a loss function L and optimizing:


$$\hat{b_{t}}={argmin}_{b_{t}\in [\text{0},\text{1}{]}^{V}}L(y_{t}-Xb_{t})$$

In the following, we use the soft $l_{\text{1}}$ loss (SL1)


$$L_{SL\text{1}}(z,\alpha )=\sum_{i}\left({{\frac{\text{1}}{\alpha }z}_{i}}^{\text{2}}\cdot \text{1}(|z_{i}|\leq \alpha )\;+\;|z_{i}|\cdot \text{1}(|z_{i}|>\alpha )\right)$$

which interpolates between the least squares (LS) loss $L_{LS}(z)=||z|{{|}_{\text{2}}}^{\text{2}}$ and the least absolute deviation loss $L_{LAD}(z)=||z|{|}_{\text{1}}$ and is a common choice in robust statistics. Here, α is a hyperparameter controlling the tradeoff between robustness and efficiency.

### Temporal regularization

Wastewater sequencing data typically displays high noise levels, possibly leading to dropouts (i.e., missing values, genomic regions with no coverage). Excluding the missing values of $y_{t}$ corresponds to removing rows of the variant design matrix X. In the worst (and common) case, this leads to the variant design matrix X to be rank deficient. In such a case, the variants relative abundances $b_{t}$ are not identifiable without further assumptions. A common regularizer in regression settings where the design matrix is singular (or has a prohibitively high condition number) is the so-called ridge penalty [19]. Here, we do not apply a ridge penalty to the magnitude of the relative abundance vectors, but further assuming temporal continuity of the variant abundances we introduce a fused ridge penalty on the difference between relative abundance vectors of different timesteps. As variants defined by more mutations contribute to more terms in the loss computation, they risk being less penalized relative to variants with fewer mutations. To avoid this, we distribute the penalization through X on the entries of $b_{i}-b_{j}$. The quadratic penalty is therefore formulated by $\lambda_{ij}||X(b_{i}-b_{j})|{{|}_{\text{2}}}^{\text{2}}$ where $\lambda_{ij}$ controls the penalization for different time differences between $b_{i}$ and $b_{j}$, such that the complete penalized loss to minimize is


$$L(b_{\text{1}},\;...,\;b_{T},\;\lambda ,\;\alpha )\;=\;\sum_{i=\text{1}}^{T}L_{SL\text{1}}(y_{t}-Xb_{t},\alpha )+\sum_{i=\text{1}}^{T}\sum_{j=\text{1}}^{T}\lambda_{ij}||X(b_{i}-b_{j})|{|}_{\text{2}}^{\text{2}}$$

In the following, we introduce a temporal decoupling of the optimization problem, allowing $b_{t}$ at each time step t to be estimated independently, for improving efficiency and numerical stability. Assuming a LS loss (i.e., SL1 with α=1) we have


$$L(b_{\text{1}},\;...,\;b_{T},\;\lambda )\;=\;\sum_{i=\text{1}}^{T}||y_{i}-{Xb}_{i}|{|}_{\text{2}}^{\text{2}}+\sum_{i=\text{1}}^{T}\sum_{j=\text{1}}^{T}\lambda_{ij}||X(b_{i}-b_{j})|{|}_{\text{2}}^{\text{2}}$$

For minimization, we compute the gradient w.r.t $b_{i}$ and set it to zero such that


$$\frac{\partial L}{\partial b_{i}}\;=\;\text{2}X^{T}Xb_{i}-\text{2}X^{T}y_{i}+\text{2}X^{T}X\sum_{j=\text{1}}^{T}\lambda_{ij}(b_{i}-b_{j})=\text{0}$$

Where we assumed, without loss of generality, that $\lambda_{ij}=\lambda_{ji}$. We define $\mu_{i}=\text{1}+\sum_{j=\text{1}}^{T}\lambda_{ij}$ to obtain


$$\mu_{i}b_{i}-\sum_{j=\text{1}}^{T}\lambda_{ij}b_{j}=\;{\left(X^{T}X\right)}^{-\text{1}}X^{T}y_{i}$$

We define the symmetric matrix


$$\Lambda =[\begin{array}{ccc} {\mu_{\text{1}}\;-\;\lambda }_{\text{1}\text{1}} & \cdots & {-\;\lambda }_{T\text{1}}\\ \vdots & \ddots & \vdots \\ {-\;\lambda }_{\text{1}T} & \cdots & {\mu_{T}\;-\;\lambda }_{TT} \end{array}]$$

If 𝛬 is full rank, which is easy to verify for example if $\lambda_{ij}>\lambda_{ik}\;\forall \;|k-i|>|k-j|$ (meaning that the penalization should encode a smoothness constraint), we obtain the solution


$$\left[\hat{b_{\text{1}}},\;...,\;\hat{b_{T}}\right]=\;{\left(X^{T}X\right)}^{-\text{1}}X^{T}\left[y_{\text{1}},\;...,\;y_{T}\right]{𝛬}^{-\text{1}}$$

With the common convention of nonnegative penalties $\lambda_{ij}$, we have that ${𝛬}^{-\text{1}}$ is also doubly stochastic. We remark that the solution is equivalent to solving the square loss solution with additional simultaneous kernel smoothing using the kernel function $k(i,j)={\left[{𝛬}^{-\text{1}}\right]}_{ij}$. Thus, Instead of specifying the $\lambda_{ij}$ penalties, we rather specify the more interpretable kernel function k(i,j), a non-negative, non-increasing function of i−j. Extending equation (1) we have:


$$\frac{\text{1}}{Z(t)}E[Y|b_{t}]k_{t}=Xb_{t}$$

where $Y\in {\left[\text{0},\text{1}\right]}^{M\times T}=\left[y_{\text{1}},...,y_{T}\right]$, $k_{t}={\left[k\left(t,t_{\text{1}}\right),...,k\left(t,t_{T}\right)\right]}^{⊤}$ and $Z(t)=\sum_{t^{\prime }\in T}k(t,t^{\prime }).\;$ In *LolliPop*, we use the Gaussian kernel $k_{Gaussian}(t,t^{\prime },\kappa )=exp\left\{\frac{-(t\;-\;t^{\prime }{)}^{\text{2}}}{\text{2}\kappa }\right\}$ with bandwidth hyperparameter κ, which is a common choice in nonparametric statistics.

### Solving the deconvolution problem

With observed mutation frequencies Y and known variant definitions X as input, we solve the deconvolution problem for a given kernel $k(t,t^{\prime })$ and loss function L(z) by finding $\hat{b_{t}}$ as


$$\hat{b_{t}}={argmin}_{b_{t}\in [\text{0},\text{1}{]}^{V}}L(\frac{\text{1}}{Z(t)}Yk_{t}-Xb_{t})$$

To numerically solve this optimization problem, we use routines from the Python scientific computing library *Scipy* [20]*.* In general we use the Trust Region Reflective method [21], but for α=1 we can switch to using the LS loss function along with Scipy’s faster non-negative Least Squares solver [22].

### Confidence intervals

To use WBE for robust decision making, it is essential to provide estimates of the uncertainty in the prediction of relative variant abundances. In the variant deconvolution step, we make assumptions only on the conditional first moment of the mutation frequencies. The corresponding least-squares estimator is a linear probability model estimator solving the estimating equations ${\;E[y}_{t}-Xb_{t}]=\text{0}$. Although it is unbiased and consistent, its default standard errors are not suitable for confidence interval construction [23]. Further assumptions are needed for computing confidence intervals, and we pursue two different strategies: one based on the bootstrap and one based on an analytical approximation of the standard errors.

It is typical to assume that observations are independent and identically distributed, implying a binomial conditional distribution of the number of positive observations (i.e., here mutated reads). However, as sequencing and amplification processes introduce strong dependencies between reads – many reads are simply copies of each other –, this assumption can lead to misspecification of the conditional variance of the fraction of mutated reads. Therefore, we here do not assume the reads to be independent in the bootstrap procedure, nor do we derive analytical standard errors under a strict binomial model.

### Bootstrapping

A popular strategy to compute confidence bands is to use the non-parametric bootstrap by resampling observations with replacement [24,25]. Here, we do not resample the individual reads due to strong dependence between them, but instead adopt a cluster bootstrap approach [26] by resampling M mutation indices from m∈1, ...,M with replacement – an approach analogous to the resampling of alignment sites in phylogenetics [27]. We do so B times to construct B bootstrap resamples of the whole time series. Each bootstrap sample is then processed by deconvolution and smoothing, resulting in B time series of dimensionality V. For each relative variant abundance v∈1, ...,V, confidence intervals are constructed at each timepoint t from the empirical quantiles of the bootstrap samples. This approach has the merit of producing confidence intervals restricted to the [0,1] range without further reparametrization.

### Asymptotic confidence intervals

We assume that at a given time t, the proportion $y_{t,m}$ of reads supporting mutation m follows a Binomial/n distribution with parameter $\pi_{m}$, i.e.,


$$p(y_{m}|\pi_{m})\propto {\pi_{m}}^{y_{m}}(\text{1}-\pi_{m}{)}^{\text{1}-y_{m}}$$

Using the linear probability model (Eq. 1), we have ${\pi_{m}=[Xb]}_{m}$. We additionally assume conditional independence of the mutation proportions such that $p(y\;|\;\pi )=\prod_{m\in M}p(y_{m}\;|\;\pi_{m})$. We thus obtain the log-likelihood:

$l(b_{\text{1}},\;...,\;b_{M})=\sum {\mathrm{\backslash nolimits}}_{m\in M}\left\{y_{m}\text{log}\left(\pi_{m}\right)+\left(\text{1}-y_{m}\right)\text{log}\left(\text{1}-\pi_{m}\right)\right\}$, where ${\pi_{m}=[Xb]}_{m}$

Differentiating twice the log-likelihood summands, we find the Fisher information matrix (see Text 1 in S1 Text)


$$\;I_{b}(b)=E\left[-\frac{\partial^{\text{2}}}{\partial b^{\text{2}}}\ell \left(\pi (b)\right)\right]=E\left[{{\left(\frac{\partial }{\partial b}\pi \right)}^{⊤}\left(-\frac{\partial^{\text{2}}}{\partial \pi^{\text{2}}}\ell (\pi )\right)}^{⊤}\frac{\partial }{\partial b}\pi \right]=X^{⊤}\text{diag}{\left\{\pi_{m}(\text{1}-\pi {)}_{m}\right\}}^{-\text{1}}X\;$$

We extract the asymptotic standard errors:


$${se}^{\text{2}}\left({\hat{b}}_{v}\right)\approx {\left[I{\left(\hat{b}\right)}^{-\text{1}}\right]}_{vv}$$

which are then used to construct Wald confidence intervals [28]. Here, a pseudofraction is added to the entries of b to avoid division by zero when computing the asymptotic standard errors.

### Logit reparametrization

To ensure that the confidence bands stay confined to the [0,1] interval, we compute the asymptotic standard errors and Wald confidence intervals on the logit scale $\phi_{v}=\text{logit}\left(b_{v}\right)=\text{log}\left(\frac{b_{v}}{\text{1}-b_{v}}\right)$, before projecting them back to the linear scale. We compute the inverse of the Fisher information matrix of ϕ by using the Delta method,


$$I_{\phi }(\phi {)}^{-\text{1}}={D_{\phi }(b)I}_{b}(b{)}^{-\text{1}}D_{\phi }(b{)}^{⊤}$$

where $D_{\phi }(b)$ is the Jacobian of the transformation, such that


$$D_{\phi }(b{)}_{ij}=\frac{\partial \phi (b{)}_{i}}{\partial b_{j}}={(-\text{1}{)}^{\text{1}(i\neq j)}\left(b_{i}\left(\text{1}-b_{i}\right)\right)}^{-\text{1}}$$

Here again, a pseudofraction is added to the entries of b to avoid division by zero.

### Overdispersion

In the likelihood model described here, taking n as the read depth can lead to a model not capturing the dispersion of the data correctly, due to reads not being independent. We thus follow an approach analogous to quasilikelihood, making more flexible assumptions on the conditional variance of the data [29]. We fix n=1 for each genomic position, and we compute the ratio of observed versus expected average square deviations of the observed data from the fitted values. This ratio is then taken as an overdispersion (or underdispersion) factor. At a given time *t*, the Wald confidence intervals are adjusted by adjusting the asymptotic s*t*andard errors for $b_{t}$:


$${\text{s}{\text{e}}_{\text{adj}}}^{\text{2}}\left({\hat{b}}_{t,v}\right)={{\sigma_{t,v}}^{\text{2}}\text{ se}}^{\text{2}}\left({\hat{b}}_{t,v}\right)$$

We build on the moment-based estimator of the dispersion factor for generalized linear models [29]


$${\sigma_{t,v}}^{\text{2}}=\frac{\text{1}}{\sum_{t^{\prime }\in T}\kappa (t,t^{\prime })}\sum_{t^{\prime }\in T}\frac{\kappa (t,t^{\prime })}{\sum_{m\in M}X_{m,v}}\sum_{m\in M}\frac{(y_{t^{\prime },m}-\hat{y_{t,m}}{)}^{\text{2}}}{\hat{y_{t,m}}(\text{1}-\hat{y_{t,m}})}X_{m,v}$$

### Implementation and availability

The methods we present here are implemented in the Python package *LolliPop,* which takes as input a tabular file of observed mutation frequencies and variant definitions, performs simultaneous kernel smoothing and deconvolution using numerical optimization, and produces confidence intervals. *LolliPop* is available on Github (https://github.com/cbg-ethz/lollipop) and as a Bioconda package. *LolliPop* is available within *V-pipe 3.0* [30]*.* All data and code used to produce the results presented in this article is available at https://doi.org/10.5281/zenodo.15277338.

### Processing of wastewater sequencing data

We used the wastewater sequencing data from the Swiss surveillance project reported in [8]. The dataset contains 1295 NGS datasets from longitudinal samples collected at six major WWTPs in Switzerland, sampled daily between January 2021 and September 2021. In brief, 24-hour raw influent composite samples were submitted to filtering, total nucleic acid extraction and reverse transcription. SARS-CoV-2 was amplified using the ARTIC v3 protocol [31], which amplifies almost the whole viral genome using 98 amplicons of roughly 400 bp each, before being submitted to NGS. The data were processed using *V-pipe 3.0* [30]*.* We defined the variants of concern (VOCs) B.1.1.7 (Alpha), B.1.351 (Beta), P.1 (Gamma), B.1.617.1 (Kappa), and B.1.617.2 (Delta) by querying the mutations present in ≥80% of the clinical sequences defining these variants on Cov-Spectrum [32] and supported by at least 100 clinical sequences. We then called these mutations in the wastewater samples from pileups of the read alignments. We defined the lineage “other” as the complement of all the mutations in this set of VOCs (i.e., a profile with no mutations). We deconvolved using different hyperparameter values (see below). We computed Wald confidence intervals adjusted for overdispersion with logit reparametrization, as well as bootstrap-based confidence intervals (1000 bootstrap samples).

### Comparison to clinical data

Using the LAPIS API of Cov-Spectrum [32], we retrieved counts of sequenced SARS-CoV-2 PCR-positive clinical samples for Switzerland, stratified by submitting lab, canton, and inferred variant. We restricted the data to samples from the large clinical testing company Viollier, where the PCR-positive samples are randomly subsampled before being sent for sequencing. We compare each WWTP to the clinical data from the canton it is located in. For the Berne WWTP of Laupen, we compare to an aggregate of the clinical sequences from both the cantons of Bern and Fribourg, as the catchment area is split between those two cantons [8]. For comparing the clinical and wastewater time series, we computed their lagged cross-correlations for time lags ranging from 30 days of lead time for the clinical signal to 30 days of lead time for the wastewater signal. In each case, we computed the overall cross-correlation $R^{\text{2}}$ as well as the per location $R^{\text{2}}$, both weighted by the square root of the clinical sample size.

### Simulations

As a matrix inverse problem, the variant deconvolution problem can be sensitive to collinearity in the variant definition matrix, which is determined by the genetic similarity between variants and exacerbated by noise and missing values. To assess the robustness of *LolliPop* to the degree of similarity between variants and the fraction of missing value in the data, we simulated wastewater sequencing data for a range of different scenarios. For timesteps t∈1,...,T and variants v∈1,...,V, we generated deterministic time series of variant relative abundances from a multinomial logistic growth model


$$b_{t,v}=\frac{\text{exp}\{a_{v}(t-m_{v})\}}{\sum_{v^{\prime }}\text{exp}\{a_{v^{\prime }}(t-m_{v^{\prime }})\}}$$

where the mixing of variants is controlled by parameter vectors a and m, which control the fitness and introduction times of the different variants. The expected frequencies of mutations at time t are computed as


$${y^{s}}_{t}=\frac{Xb_{t}+\delta }{\text{1}+\text{2}\delta }$$

where X is the variant definition matrix and δ denotes the per-base error probability in the sequencing process. The read depth at position m and time t is then sampled as


$$n_{m,t}\sim \text{NegativeBinomial}(\mu ,\;\sigma_{\text{1}})$$

where μ and $\sigma_{\text{1}}$ are the expected value and overdispersion parameter, respectively, of the read depth in covered regions. To simulate levels of dropouts higher than expected from this model we further randomly set entries $n_{m,t}$ to zero with probability p. The observed mutation frequency at position m and time t is then sampled as


$${y^{o}}_{m,t}\sim \text{BetaBinomial}({y^{s}}_{m,t},\;\sigma_{\text{2}},\;n_{m,t})/n_{m,t}$$

with expected value ${y^{s}}_{m,t}$ and overdispersion parameter $\sigma_{\text{2}}$.

### Simulation of Delta taking over Alpha

To assess the efficacy of our method to track the important situation where a new variant displaces the currently circulating one over time, we generated a 60-timestep time series of Delta taking over Alpha at the logistic growth rate of 0.1/day. We used the variant definitions generated from *Cov-Spectrum* [32] using the toolkit from *COJAC* [8]. We set μ=1000, $\sigma_{\text{1}}=\text{1}\text{0}$, δ=0.0025 and $\sigma_{\text{2}}=\text{1}\text{0}\text{0}\text{0}$. To assess the robustness of our method to varying levels of noise, we varied the dropout probability p between 0, 0.1, 0.2, …, 0.9 and 0.99. These simulated datasets were deconvolved using the SL1 (α=0.135) loss, as well as with the LS (α=1) loss to assess the robustness added by the SL1 loss. We varied the bandwidth κ of the smoothing kernel between 0, 30 and 60. We compared the deconvolved value to the known ground truth and computed their squared correlation coefficient R^2^.

### Simulation of a mixture of Omicron subvariants

To further test our method in a more complicated situation where multiple related variants co-circulate, we generated another 60-timestep time series of a mixture of highly similar Omicron subvariants BA.2, BA.5, BA.2.75, BQ.1.1 and XBB. We set the other parameters of the simulation to the same values. We deconvolved using the same parameters and compared the results to the ground truth similarly.

### Robustness to misspecification

To investigate the effect of adding variants not present in the samples (model overspecification), we generated data using the same two scenarios as above but included additional related variants in the deconvolution. For the Delta–Alpha time series, we added Beta (B.1.351) and Kappa (B.1.617.1), a close relative of Delta. For the Omicron mixture, we added BA.4 to the deconvolution.

Conversely, to assess the effect of omitting a truly circulating variant (model underspecification), we removed XBB from the deconvolution in the Omicron subvariant scenario. All simulations used identical parameter settings and noise levels as described above.

### Simulation of a mixture of artificial closely related variants

Finally, we generated an artificially adversarial 60-timestep time series of a mixture of 5 highly related variants. Their definitions were generated by taking all size 4 subsets of a set of 5 mutations. This ensures no mutation is exclusive to any single variant, but that each of them is found in 4 of the 5 variants. Thus, each pair of variants shares all but one mutation. We set the rest of the parameters to the same values as before, deconvolved using the same procedure, and assessed the results similarly.

### Hyperparameters

We assessed the sensitivity of our deconvolution method to hyperparameter choice both on simulated and on real wastewater data. For the simulated data, we assessed the root mean square error of the deconvolution of a simulated mixture of 5 Omicron subvariants across a grid of values for the smoothing bandwidth κ parameter and the α parameter (controlling the breakpoint between $l_{\text{2}}$ and $l_{\text{1}}$ loss) in (κ,α)∈[0,30] ×[0.01, 1.0], for different levels of missing data.

For the real wastewater sequencing data, we analyzed the two biggest WWTPs (Zurich and Vaud) using a similar grid of hyperparameters. For each deconvolved dataset, we linearly regressed the relative abundances of the different variants in clinical sequencing on the relative abundances in wastewater inferred by the deconvolution, using the statistical software *R* [33], and we reported the R^2^. The regressions were performed with data points weighted by the square root of the clinical sample sizes.

## Results

**Fig 1:**
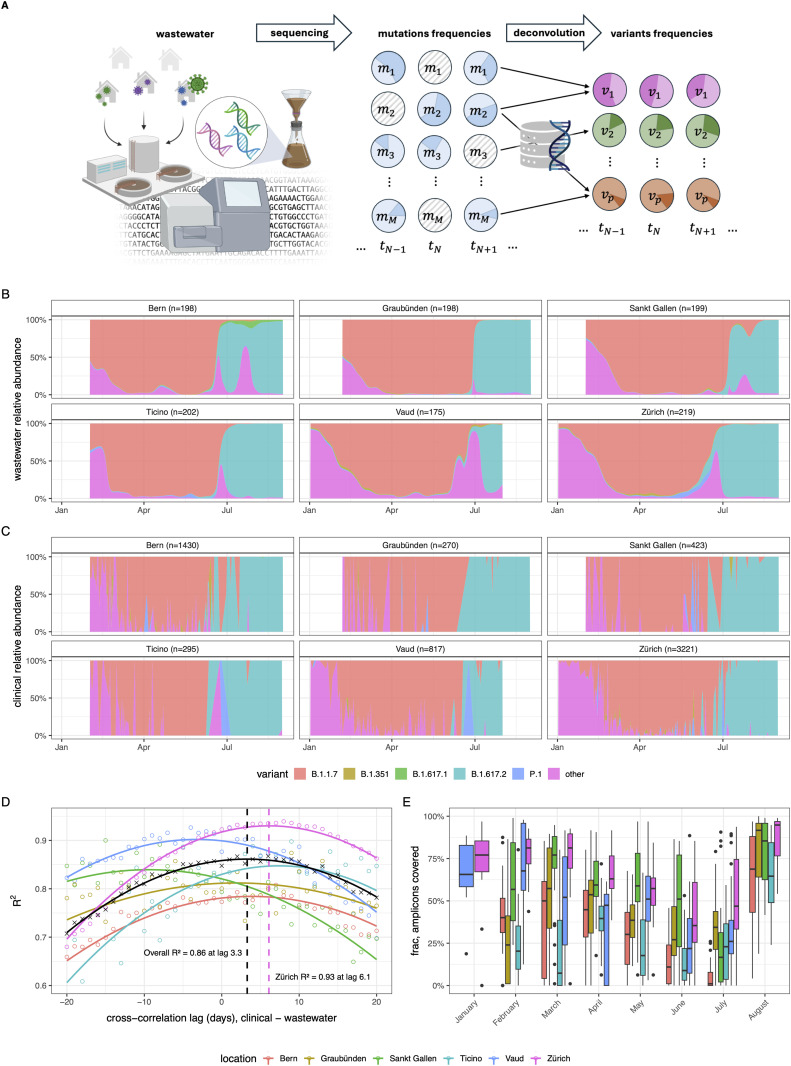
Overview of *LolliPop* for variant deconvolution. **A** Mutation frequencies in wastewater samples of different timepoints are obtained from NGS read counts. The mutation frequencies are deconvolved using the variant definitions, producing estimates of relative variant abundances along with confidence intervals. Repeating the operation for each timepoint tracks the relative abundances of the variants through time. Some values for mutation frequencies are missing (gray), but temporal regularization allows for deconvolution with high levels of missing data. Created in BioRender. Dreifuss, **D.** (2025)*.* **B** Estimates of variant relative abundances obtained from deconvolution of wastewater data in different WWTPs. Different colors correspond to the different genomic variants studied. The deconvolution was performed with a Gaussian smoothing kernel with κ=30 and a scale parameter α=0.135. **C** Relative abundances of variants in clinical samples of the cantons surrounding the studied WWTPs **D** Cross-correlation between the wastewater deconvolved values of relative abundances and the clinical data estimates, overall (black) and for the different locations (colors). The correlation values are computed weighted by $\sqrt{n_{clinical}}$. Dots mark measured cross-correlations at different lag times, lines mark quadratic fits. The overall cross-correlation peaks at $R^{\text{2}}=\text{0}.\text{8}\text{6}$ for a wastewater signal lead time of 3.3 days. **D** Fraction of amplicons covered in the wastewater sequencing data, for each month and for each location (colors). The coverage drops drastically for periods with low incidence (see Fig A in S1 Text). The central line within each box represents the median coverage per month, while the box itself spans the monthly interquartile range (IQR). The whiskers extend to the most extreme data points still within 1.5 times the IQR, and data points beyond this range are shown as individual dots.

We developed *LolliPop*, a statistical and computational method for deconvolving variant abundances from wastewater sequencing data (Fig 1A). It estimates relative abundances variants from observed mutation frequencies using a variant profile matrix, a flexible loss function, and temporal regularization. Below, we compare its performance on Swiss wastewater data against matched clinical estimates and evaluate its robustness on simulated datasets with high levels of missing data.

### Comparison to clinical data

We compared time series of relative variant abundances inferred from wastewater sequencing data using *LolliPop* to those estimated using clinical data. To challenge the robustness of *LolliPop*, we used wastewater data including samples from a low incidence period of the pandemic, where viral concentrations were extremely low (Fig A in S1 Text). The cumulative aligned reads depth per sample ranged between 0 and 17,369,931, with a median of 239,812, a mean of 1,315,240 and a standard deviation of 2,583,714. Only 9 samples had full coverage, with genome coverage dropping extremely low during periods of low incidence (Figs 1E, Fig A in S1 Text). Despite this very high level of missing data, it was still possible to deconvolve the observed mutations into variant relative abundances accurately (Fig 1). We found that the wastewater-based infection dynamics closely follow those derived from clinical sequencing (Fig 1B,1C).

Due to the delay distribution between infection and clinical testing not necessarily matching the delay distribution of shedding and sewage travel time, we expect that the signal in wastewater can be shifted in time compared to clinical samples. The overall highest cross-correlation value (weighted by root clinical sample size) between wastewater-derived estimates and clinical estimates was $R^{\text{2}}=\text{0}.\text{8}\text{6}$ estimated, with wastewater having a lead time of 3.3 days (Fig 1D). In the region of Zürich – which had both the largest clinical sample (n=3,221) and the least impacted wastewater sequencing coverage – the wastewater signal showed a lead time of 6.1 days with $R^{\text{2}}=\text{0}.\text{8}\text{6}$. We performed the deconvolution with kernel bandwidth κ=30 and a loss scale parameter α=0.135.

### Confidence intervals

**Fig 2:**
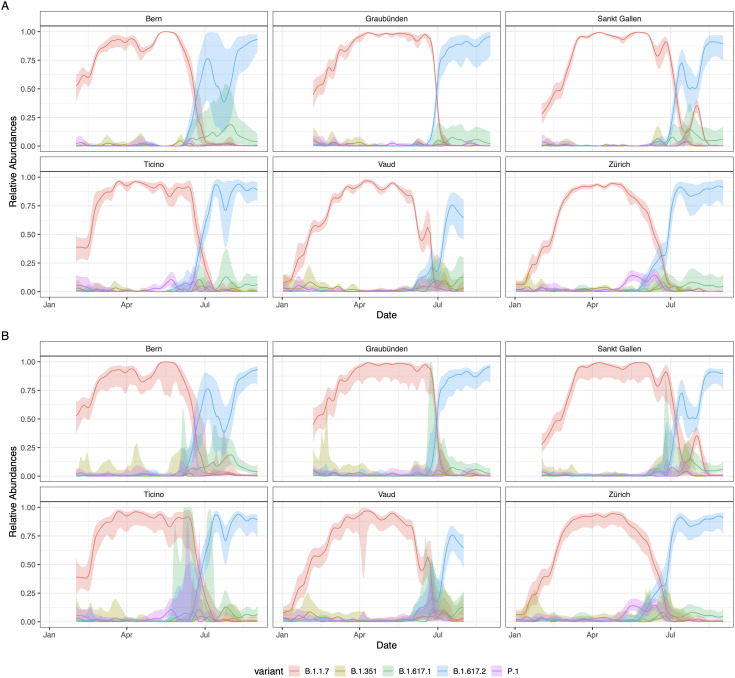
Confidence bands of the deconvolved relative abundance values, at 95% level. **a** Bootstrap confidence intervals computed using 1000 bootstrap samples of the mutations. **b** Wald confidence bands computed using the overdispersion adjusted asymptotic standard error on the logit scale, back-transformed to the linear scale.

Bootstrapping the mutation counts with subsequent deconvolution was used to produce confidence intervals of the variant relative abundances (Fig 2A). Alternatively, we also used Wald-type confidence intervals computed on the logit scale, adjusted for dispersion and then back transformed (Fig 2B). Confidence bands from both methods had great overlap – on average, Wald confidence intervals covered ~89% of the bootstrap confidence intervals –, although Wald confidence intervals seemed more conservative (Fig B in S1 Text). Especially in the Wald confidence intervals, uncertainty was consistently higher during the months in which wastewater samples contained low concentrations of SARS-CoV-2 RNA (June-July) due to low incidence of the virus (Figs 1D, Fig A in S1 Text).

### Simulations

**Fig 3:**
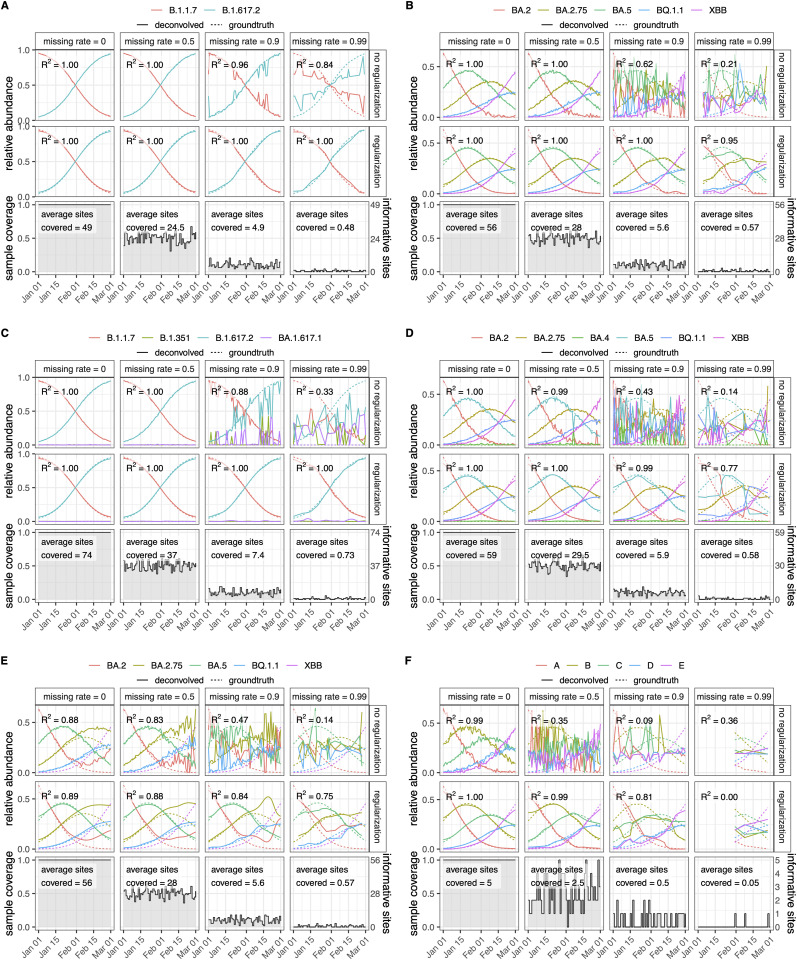
Simulation experiments assessing robustness to missing data, variant similarity, and model misspecification. Each panel shows a 60-day simulated time series of variant competition. Within each panel, columns correspond to increasing rates of missing data (0%, 50%, 90%, 99%). The first and second rows display deconvolution results without and with temporal regularization, respectively. Dashed lines indicate the simulated ground truth, while solid lines show deconvolution results obtained with the SL1 (=0.13) loss function. Annotations report the R² values compared to ground truth. The third row shows the coverage of informative sites (i.e., the fraction of non-missing data) for each simulated sample. See Fig C in S1 Text for an analysis of the similarity between the variant profiles used in the simulations. **A** Simulated time series of B.1.617.2 (Delta) overtaking B.1.1.7 (Alpha). **B** Simulated time series of five highly similar Omicron subvariants. **C** Same as **(A)**, but with two additional related variants included in the deconvolution (overspecified model). **D** Same as **(B)**, but with one additional related variant included in the deconvolution (overspecified model). **E** Same as **(B)**, but omitting XBB from the deconvolution (underspecified model). **F** Simulated mixture of five artificial highly related variants generated from all subsets of size four out of five informative sites.

The variant deconvolution is sensitive to the similarity of variants as well as to the amount of missing values (i.e., low coverage) in the sequencing data. To test the limits of the robustness of *LolliPop*, we simulated data from mixtures of variants with varying degrees of similarity (Fig C in S1 Text) and missing data. In all simulations, increasing the level of missing values eventually prevented the deconvolution to recover the signal in the absence of regularization (Fig 3, Fig D in S1 Text). However, we found that temporal regularization allowed the relative abundances to be estimated accurately (R^2^ > 0.9), even in the case of very high levels (>90%) of missing data.

When considering the prototypical case of a divergent variant (Delta) sweeping over the dominant strain (Alpha), the original signal was recovered with high accuracy (R^2^ > 0.99) despite up to 99% of missing values (Fig 3A, Fig D.A in S1 Text, Fig E in S1 Text). When tracking multiple co-circulating closely related lineages (Omicron derivatives), the original signal could also be recovered with high accuracy (R^2^ = 0.95) with up to 99% missing values (Fig 3B, Fig F in S1 Text). When the lineages were further made as similar as possible, *LolliPop* could still recover the signal accurately (R^2^ = 0.92) despite up to 90% of missing values – i.e., an average of only 0.5 informative sites covered in each sample (Fig 3F, Fig D.F in S1 Text, Fig G in S1Text). In contrast to temporal regularization, switching from the LS to the SL1 loss had only a minor effect on robustness to missing data. Int all simulated scenarios, it provided a slight improvement in stability, which was noticeable only in the absence of regularization.

Next, we investigated the impact of model misspecification by either including variants not present in the simulated samples (overspecified model) or omitting existing variants from the deconvolution (underspecified model). Model overspecification (Figs 3C,3D, Fig D.C,D in S1 Text) had no noticeable effect in the case of medium or high coverage. However, under low coverage, it led to sporadic false-positive detections of variants closest related to the ones in circulation. In contrast, model underspecification (Figs 3E, Fig D.E in S1 Text) systematically caused the abundance of the omitted variant to be misattributed to one or more related variants. In the absence of temporal regularization, this was visible from a noisier deconvolved signal.

In all cases, the smoothing effect of temporal regularization introduced some bias at the start and end of the time series, a well known artifact of kernel smoothers [34]. The bias introduced by smoothing was generally small, and in the presence of missing values the added error was by far compensated by the reduced variance of the estimates. In the case of high relative noise, the temporal regularization offered added accuracy even in the absence of missing data, by acting as a smoother (Fig 3B, Fig D.B in S1 Text, Fig G in S1 Text).

### Hyperparameters

We ran *LolliPop* on simulated data consisting of a mixture of five closely related Omicron subvariants, using a grid of hyperparameter values. These tests were performed under varying levels of missing data. Increasing the smoothing bandwidth κ from zero initially reduced the RMSE, but it gradually increased again at higher values due to bias introduced by oversmoothing (Fig H in S1 Text). The benefit of using a nonzero smoothing bandwidth was greater in the presence of high levels of missing data. In contrast, the choice of the loss scale parameter α (which controls the breakpoint between $l_{\text{2}}$ and $l_{\text{1}}$ loss) had a negligible effect on RMSE.

Next, we applied *LolliPop* to the Swiss wastewater monitoring data from the two largest WWTPs, again using a grid of hyperparameter values. Increasing the smoothing bandwidth κ improved the goodness of fit with clinical sequencing data, as measured by R^2^ (Fig I in S1 Text). For the loss scale parameter α, the best fits were obtained for values between 0.1 and 0.25.

### Runtime

Wall time on a standard laptop (MacBook Air M1 2020, max clock rate 3.2 GHz, 16Gb RAM) was ~ 2s for deconvolving all 1295 wastewater samples (225 timepoints, 6 locations, 5 variants defined through 94 mutations) using the LS loss function (i.e., SL1 with α=1). Using the SL1 loss function, wall time was ~ 1min for the same task. Wall time was ~ 1min for reparameterized Wald confidence intervals. Constructing confidence intervals from 1000 bootstrap samples) had a wall time of ~30min. All computations were performed using a single CPU core.

## Discussion

We have presented *LolliPop*, a method for solving the variant deconvolution problem. Deconvolving patterns of mutations in wastewater NGS data is necessary to track the relative abundance of viral variants. We showed its application to data from the Swiss wastewater monitoring program, extending over eight months at six different locations around the country. We found that the deconvolved values of relative abundance closely follow the dynamics observed in the clinical sequencing effort, even when the coverage was very low. We further evaluated the robustness to missing values of our method on simulated time series of increasing complexity. We found that ground truth values can be recovered with high accuracy in the presence of very high rates of missing values.

*LolliPop* takes two main tunable hyperparameters. The most important one is the bandwidth κ controlling the amount of temporal regularization and smoothing. Setting κ=0, *LolliPop* will deconvolve each sample independently. Samples with coverage dropouts might fail to deconvolve, or yield unstable estimates of variant relative abundances. In our simulations and empirical data, we have seen that setting κ to even a small non-zero value (like κ=5 days, for a daily sampled time series) can make the deconvolution vastly more robust and increase its accuracy. For datasets with lower sampling frequency or when stronger smoothing is desired, the value of κ can be increased, though at the cost of increased computation time. The second hyperparameter is the loss scale α, which controls the influence of mutation frequency outliers. In our analyses, the influence of this hyperparameter on the accuracy of the deconvolution was minor compared to κ. For computational efficiency, we recommend using the LS loss (i.e., α=1), and for marginal increase in robustness and accuracy we recommend using values of α between 0.1 and 0.25.

In this manuscript we focused on SARS-CoV-2, but the method is broadly applicable to other pathogens. *LolliPop* relies solely on observed mutation frequencies in mixed samples and predefined variant profiles, making it readily adaptable to different pathogens and surveillance contexts. Notably, *LolliPop* was recently applied to Influenza A, where it successfully deconvolved wastewater sequencing data from Switzerland during the 2023–2024 season into the relative abundances of the major H1N1 clades. These estimates showed strong agreement with matched clinical data [35]. Additionally, *LolliPop* was successfully applied to Respiratory Syncytial Virus (RSV), where it was used to deconvolve RSV-A and RSV-B wastewater sequencing data from Switzerland during the 2023–2024 season into the relative abundances of their major lineages, enabling the identification of temporal and regional patterns [36].

We have shown that *LolliPop* is computationally efficient, capable of processing thousands of samples within seconds or minutes on a standard laptop using a single CPU core. This efficiency makes it suitable and readily applicable to large-scale analyses, including national wastewater surveillance efforts such as the Swiss wastewater monitoring program (https://wise.ethz.ch).

A broader limitation of *LolliPop* is that it relies on predefined variant profiles to perform the deconvolution. These variant definitions are typically derived from clinical sequencing repositories. In principle, the variant profiles could also be inferred directly from wastewater using additional analytical tools, although this is currently not reliably possible. As a consequence, emerging variants cannot be accurately tracked until their characteristic mutation profile becomes available from external data. In practice, even a single clinical genome of a new variant – potentially from another country – is sufficient to define its haplotype and enable wastewater-based tracking. *LolliPop* is therefore most effective when embedded within an integrated genomic surveillance system.

Variant selection is a critical step for the deconvolution. Although a large number of SARS-CoV-2 variants have emerged, typically only a few circulate at any given time. We have shown in simulations that overspecifying the deconvolution can lead to false positives when the coverage is low. Selecting the appropriate set of variants to deconvolve the signal into is therefore essential for accurate inference. For this purpose, the more sensitive and selective tool *COJAC* [8] can be used to confirm variant presence prior to deconvolution. *COJAC* is integrated within the *V-pipe* [30] framework alongside *LolliPop*, facilitating streamlined analysis.

Another limitation is that the current implementation considers genomic positions independently, which can lower the sensitivity of the detection for very low abundance variants. However, local haplotype information is often available, for example from read-pairs profiles. *LolliPop* could in principle accept local haplotype counts instead of mutation counts as an input to the deconvolution, with variants being defined from their local haplotype profiles instead of mutation profiles.

Modifications to *LolliPop* can be easily implemented. First, the behavior of the deconvolution under different types of loss functions could be investigated. We have used here the Soft $l_{\text{1}}$ loss, and the results were not very strongly affected by the choice of the scale parameter α, indicating robustness of the method to this choice. Other types of losses could certainly have interesting properties for the problem at hand. For example, if the number of candidate lineages to deconvolve grows, building in a sparsity assumption by adding an $l_{\text{1}}$ regularization term of the relative variant abundances could lower the variance of their estimates.

Another component that can readily be modified is the type of kernel used for enforcing temporal regularization. We have used a Gaussian kernel, but other choices could be relevant depending on the application. For example, kernels elicited on expert knowledge could be used for more precisely relating the relative abundances in samples to the relative incidences in the population. The temporal dynamics of viral shedding in wastewater are generally described by a shedding load distribution [37], which could be accounted for by an asymmetric and non zero-centered kernel. In general, we recommend using a kernel with sufficient bandwidth to robustify the method against missing values. However, temporal variability between samples does not always reflect noise or sampling error: at certain locations, such as small catchments, sharp changes in variant composition may represent true epidemiological signals. In such cases, users should avoid oversmoothing real temporal dynamics.

To assess the uncertainty in the estimates of relative abundances, we have derived a bootstrap-based and an analytical approach to produce confidence intervals. Bootstrap confidence intervals are conceptually straightforward to derive but are by design computationally intensive, whereas analytical expressions for confidence intervals can provide substantial speedup. The Wald confidence intervals we derived here are based on a binomial/quasibinomial variance structure, and include three components of the variance of the estimates: one to account for the mutation overlap between variant definitions, one to account for the quadratic form of the variance of a binomial sampling, and one to account for overdispersion in read counts. The Wald confidence intervals computed on the linear scale suffers from known shortcomings, as it can exit the [0,1] range, which is why we compute them on the logit scale. In our results, the Wald confidence intervals very closely resemble the bootstrap confidence intervals, while providing an almost 30x speedup in computation time. The discrepancy between both approaches was higher when one variant was dominating and the others low in relative abundance. In that case, the resampling scheme in the bootstrap provides low variability, while the logit-transformed standard errors can become very large.

*LolliPop* has built in the assumption of temporal continuity of the variant relative abundances in the form of kernel smoothing simultaneously with deconvolution, which enforces a ridge penalty on the temporal variation of the relative abundance. As in a monitoring program, usually multiple locations are being monitored, another useful assumption to build in the deconvolution could be that of spatial continuity if the spatial resolution allows for it. Jointly smoothing the different locations might offer increased robustness to the estimates by partial pooling of the information.

To summarize, *LolliPop* solves the variant deconvolution problem, taking into account the time series nature of wastewater sequencing datasets and mitigates the extremely high levels of noise and dropouts these experiments typically display. Our method can estimate uncertainty using different approaches, including analytical confidence bands with short computation times. As such, *LolliPop* enables genomic variant tracking in large-scale wastewater-based epidemiology projects.

## Supporting information

S1 TextFig A: Viral loads in the wastewater samples and daily incidence in the catchment area of the treatment plants.Points are measured values and lines are 7-day median values. Data from https://sensors-eawag.ch/sars/overview.html (A). Log_10_ read depth per amplicon, per sample across the different locations. Gray values represent amplicons with zero coverage, i.e., dropouts (B). **Fig B:** Confidence bands of the deconvolved relative abundance values, at 95% level. Bootstrap and Wald-type confidence bands are displayed on the same plot. **Fig C:** profiles of the variants in this study. **A** mutation indices (rows) and variants (columns) of the filtered variant definition matrix used in this study. A cell is colored yellow if the mutation is present in the variant, and purple otherwise. **B** correlation matrix between the variant definitions, calculated using Pearson correlation. **C** number of mutations in common between each pair of variants. **D** Jaccard index between each pair of variants, calculated as the size of the intersection divided by the size of the union of the mutation sets. **Fig D:** Simulation experiments assessing robustness to missing data, variant similarity, and model misspecification. Each panel shows a 60-day simulated time series of variant competition. Within each panel, columns correspond to increasing rates of missing data (0%, 50%, 90%, 99%). The first and second rows display deconvolution results without and with temporal regularization, respectively. Solid and dashed lines show deconvolution results obtained with the LS (i.e., SL1 with =1) and SL1 (=0.13) loss functions, respectively. Annotations report the corresponding R^2^ values compared to ground truth. The third row shows the coverage of informative sites (i.e., the fraction of non-missing data) for each simulated sample. See Fig C in S1 Text for an analysis of the similarity between the variant profiles used in the simulations. **A** Simulated time series of B.1.617.2 (Delta) overtaking B.1.1.7 (Alpha). **B** Simulated time series of five highly similar Omicron subvariants. **C** Same as (A), but with two additional related variants included in the deconvolution (overspecified model). **D** Same as (B), but with one additional related variant included in the deconvolution (overspecified model). **E** Same as (B), but omitting XBB from the deconvolution (underspecified model). **F** Simulated mixture of five artificial highly related variants generated from all subsets of size four out of five informative sites. **Fig E:** Simulation experiments, displaying the effect of kernel smoothing on varying levels of missing value on a 60-timesteps timeseries of B.1.617.2 (de) taking over B.1.1.7. (al). Columns are different values of the smoothing bandwidth, rows are different levels of missing values. Dashed lines represent ground truth, solid lines and dotted lines show deconvolution results with the LS and SL1 loss functions, respectively. **Fig F:** Simulation experiments, displaying the effect of kernel smoothing on varying levels of missing value on a 60 timesteps time series of the five closely related Omicron derivatives BA.2 (om2), BA.2.75 (om275), BA.5 (om5), BQ.1.1 (ombq11) and the recombinant XBB (omxbb). Columns are different values of the smoothing bandwidth, rows are different levels of missing values. Dashed lines represent ground truth, solid lines and dotted lines show deconvolution results with the LS and SL1 loss functions, respectively.**Fig G:** Simulation experiments, displaying the effect of kernel smoothing on varying levels of missing value on a 60 timesteps time series of the five artificially closely related variants. Columns are different values of the smoothing bandwidth, rows are different levels of missing values. Dashed lines represent ground truth, solid lines and dotted lines show deconvolution results with the LS and SL1 loss functions, respectively. **Fig H:** Root Mean Square Error (RMSE) of the deconvolved simulated data, as a function of the kernel bandwidth κ and α scale parameters of the robust regression. The accuracy of the deconvolution was measured on the simulated data for 5 closely related omicron subvariants shown in Fig F in S1. **Fig I:** Goodness of fit of the robust kernel deconvolution of the wastewater NGS, as a function of the kernel bandwidth κ and α scale parameters of the robust regression. Goodness of fit is evaluated by regressing on estimates of relative abundances of variants obtained from clinical sequencing data.(PDF)
