# Supplementary material for: Tracking SARS-CoV-2 genomic variants in wastewater sequencing data with LolliPop
Source: PLoS Comput Biol. 2026 Feb 19;22(2):e1014003. doi: 10.1371/journal.pcbi.1014003 (PMC12945317; doi:10.1371/journal.pcbi.1014003)
Supplement: S1 Text — Points are measured values and lines are 7-day median values. Data from https://sensors-eawag.ch/sars/overview.html (A). Log10 read depth per amplicon, per sample across the different locations. Gray values represent amplicons with zero coverage, i.e., dropouts (B). Fig B: Confidence bands of the deconvolved relative abundance values, at 95% level. Bootstrap and Wald-type confidence bands are displayed on the same plot. Fig C: profiles of the variants in this study. A mutation indices (rows) and variants (columns) of the filtered variant definition matrix used in this study. A cell is colored yellow if the mutation is present in the variant, and purple otherwise. B correlation matrix between the variant definitions, calculated using Pearson correlation. C number of mutations in common between each pair of variants. D Jaccard index between each pair of variants, calculated as the size of the intersection divided by the size of the union of the mutation sets. Fig D: Simulation experiments assessing robustness to missing data, variant similarity, and model misspecification. Each panel shows a 60-day simulated time series of variant competition. Within each panel, columns correspond to increasing rates of missing data (0%, 50%, 90%, 99%). The first and second rows display deconvolution results without and with temporal regularization, respectively. Solid and dashed lines show deconvolution results obtained with the LS (i.e., SL1 with =1) and SL1 (=0.13) loss functions, respectively. Annotations report the corresponding R2 values compared to ground truth. The third row shows the coverage of informative sites (i.e., the fraction of non-missing data) for each simulated sample. See Fig C in S1 Text for an analysis of the similarity between the variant profiles used in the simulations. A Simulated time series of B.1.617.2 (Delta) overtaking B.1.1.7 (Alpha). B Simulated time series of five highly similar Omicron subvariants. C Same as (A), but with two additional related vari [file pcbi.1014003.s001.pdf]

Supplementary Material for “Tracking SARS-CoV-2 genomic variants in wastewater sequencing data with *LolliPop*”

Text 1

Let b be the vector of variant relative abundances, π be the vector of expected fractions of mutated reads given a design matrix X, i.e., $\pi(b) = Xb$. The Hessian matrix of the deconvolution likelihood model is found as follows. We differentiate twice the log-likelihood of the model

$$\frac{\partial}{\partial b}\ell(\pi) = \frac{\partial}{\partial \pi(b)}\ell(\pi(b))\frac{\partial}{\partial b}\pi(b)$$

$$\Rightarrow \frac{\partial^2}{\partial b^2}\ell(\pi(b)) = \frac{\partial}{\partial b}\left\{\frac{\partial}{\partial \pi(b)}\ell(\pi(b))\right\}\frac{\partial}{\partial b}\pi(b) + \underbrace{\frac{\partial^2}{\partial b^2}\pi(b)\frac{\partial}{\partial \pi(b)}\ell(\pi(b))}_{\pi(b)=Xb\Rightarrow\frac{\partial^2}{\partial b^2}\pi(b)=0}$$

$$= \frac{\partial}{\partial b}\frac{\partial}{\partial \pi(b)}\ell(\pi(b))\frac{\partial}{\partial b}\pi(b) \quad = \left(\frac{\partial}{\partial \pi(b)}\frac{\partial}{\partial b}\ell(\pi(b))\right)^{\top}\frac{\partial}{\partial b}\pi(b)$$

$$= \left(\frac{\partial}{\partial \pi(b)}\frac{\partial}{\partial \pi(b)}\ell(\pi(b))\frac{\partial}{\partial b}\pi(b)\right)^{\top}\frac{\partial}{\partial b}\pi(b) \quad = \left(\frac{\partial}{\partial b}\pi(b)\right)^{\top}\underbrace{\left(\frac{\partial^2}{\partial \pi(b)^2}\ell(\pi(b))\right)^{\top}}_{=\frac{\partial^2}{\partial \pi(b)^2}\ell(\pi(b))}\frac{\partial}{\partial b}\pi(b)$$

The Fisher information matrix is then

$$I_b(b) = E\left[-\frac{\partial^2}{\partial b^2}\ell(\pi)\right] = E\left[\left(\frac{\partial}{\partial b}\pi\right)^{\top}\left(-\frac{\partial^2}{\partial \pi^2}\ell(\pi)\right)^{\top}\frac{\partial}{\partial b}\pi\right] = X^{\top} diag\left\{\pi_m(1 - \pi)_m\right\}^{-1}_{m=1,\dots,M} X$$

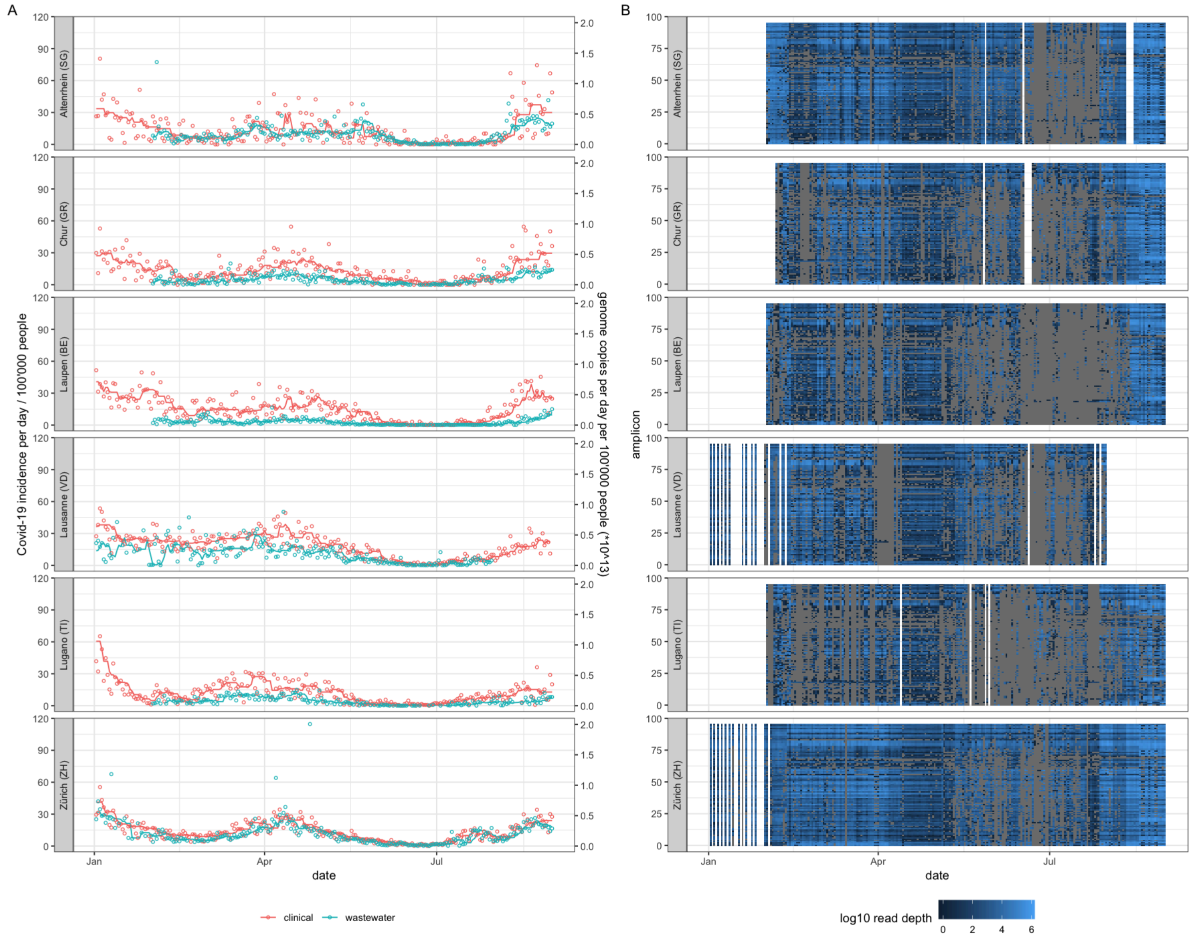

Fig A: Viral loads in the wastewater samples and daily incidence in the catchment area of the treatment plants. Points are measured values and lines are 7-day median values. Data from https://sensors-eawag.ch/sars/overview.html (A). Log_{10} read depth per amplicon, per sample across the different locations. Gray values represent amplicons with zero coverage, i.e. dropouts (B).

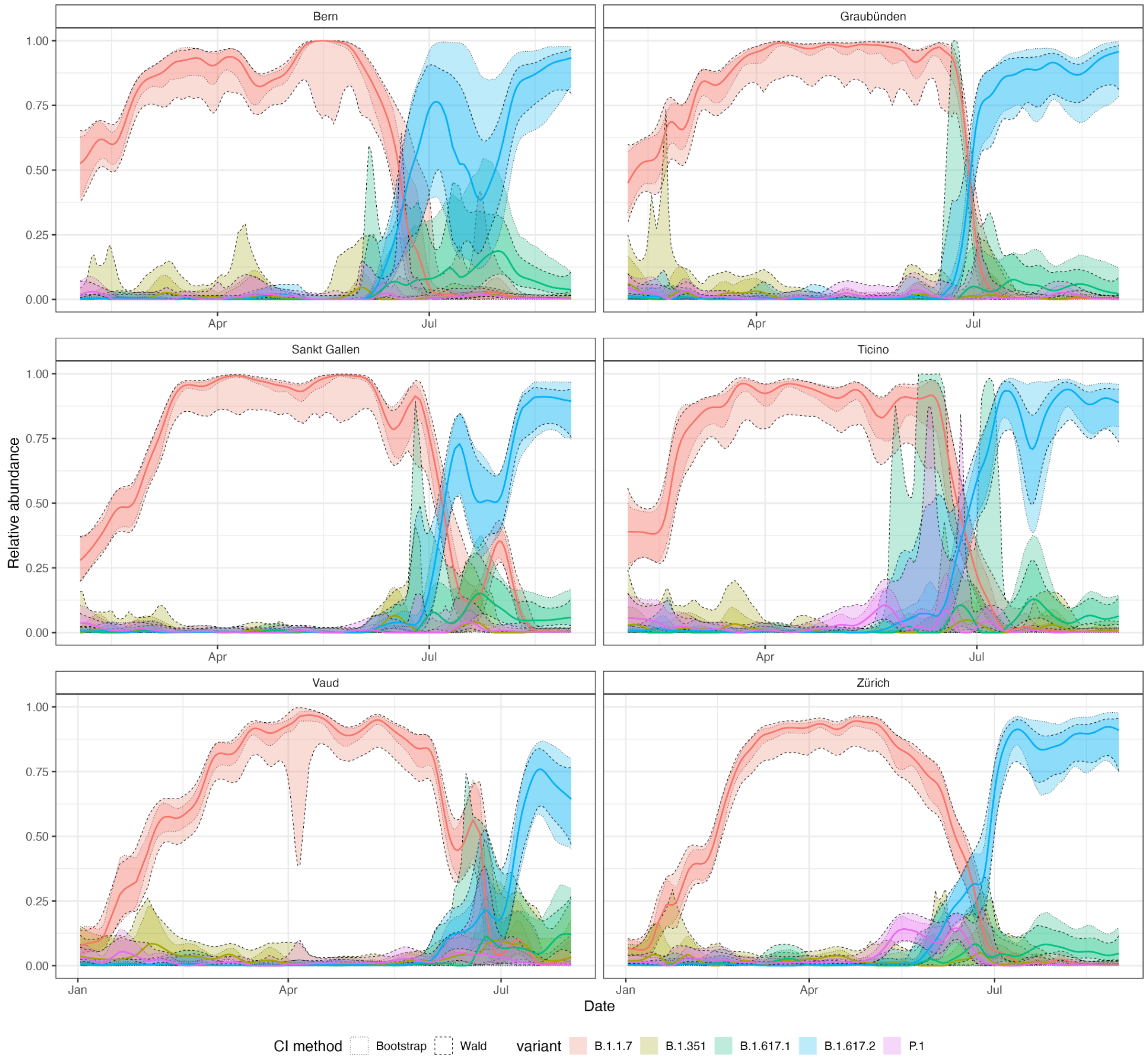

Fig B: Confidence bands of the deconvolved relative abundance values, at 95% level. Bootstrap and Wald-type confidence bands are displayed on the same plot.

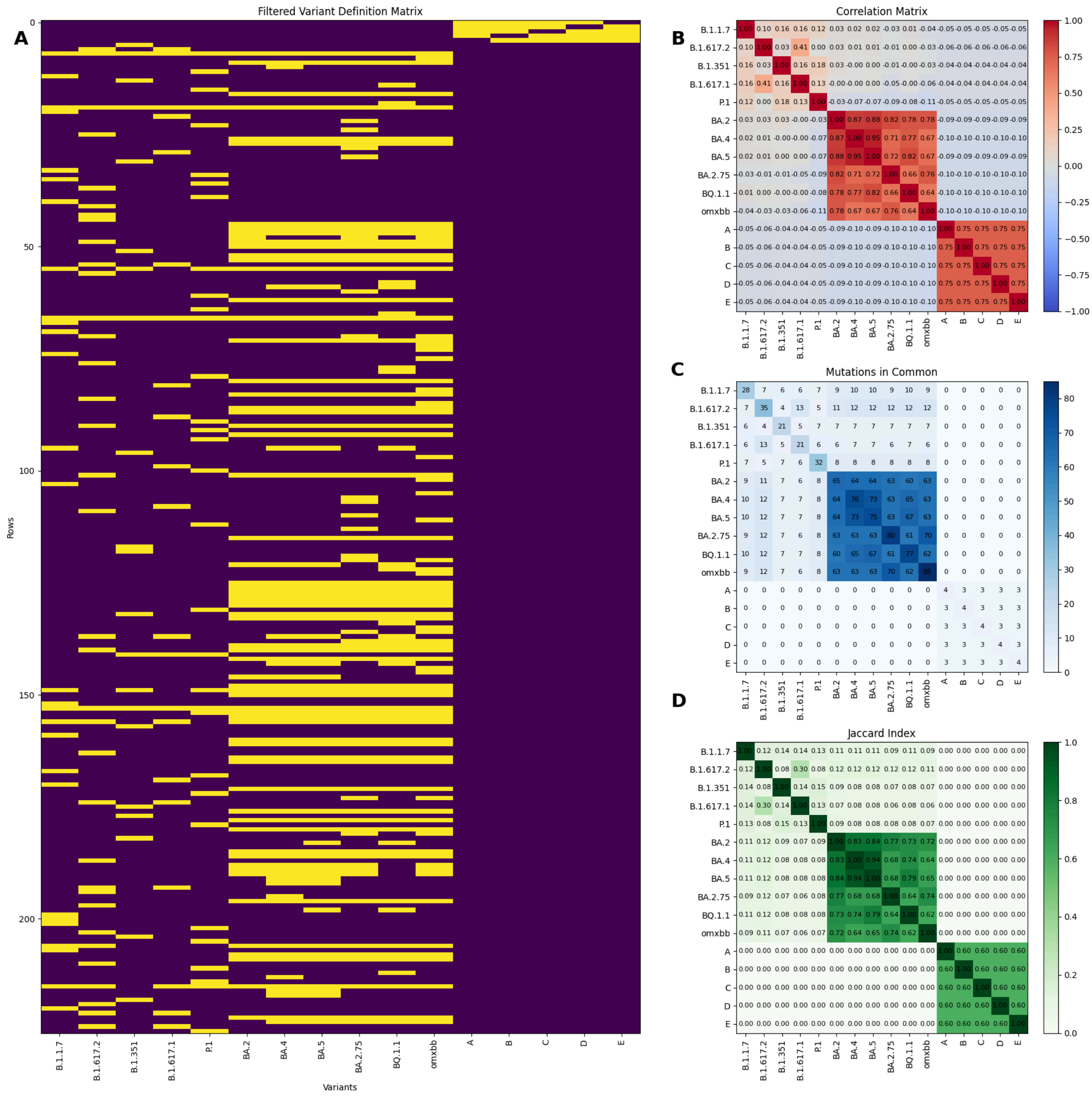

Fig C: profiles of the variants in this study. **A** mutation indices (rows) and variants (columns) of the filtered variant definition matrix used in this study. A cell is colored yellow if the mutation is present in the variant, and purple otherwise. **B** correlation matrix between the variant definitions, calculated using Pearson correlation. **C** number of mutations in common between each pair of variants. **D** Jaccard index between each pair of variants, calculated as the size of the intersection divided by the size of the union of the mutation sets.

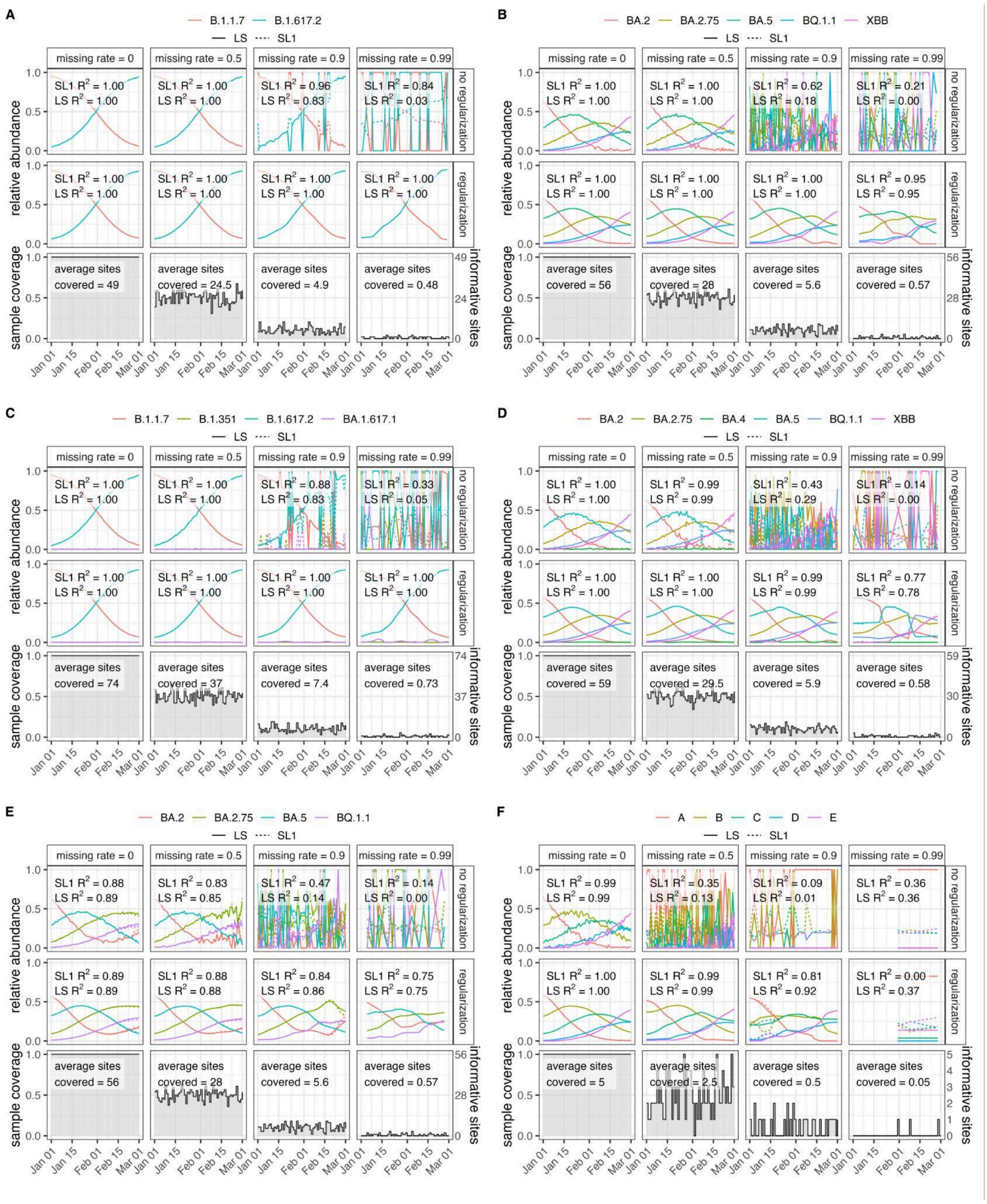

Fig D: Simulation experiments assessing robustness to missing data, variant similarity, and model misspecification. Each panel shows a 60-day simulated time series of variant competition. Within each panel, columns correspond to increasing rates of missing data (0%, 50%, 90%, 99%). The first and second rows display deconvolution results without and with temporal regularization, respectively. Solid and dashed lines show deconvolution results obtained with the LS (i.e. SL1 with $\alpha = 1$) and SL1 ($\alpha = 0.13$) loss functions, respectively. Annotations report the corresponding R^2 values compared to ground truth. The third row shows the coverage of informative sites (i.e. the fraction of non-missing data) for each simulated sample. See Fig C in S1 for an analysis of the similarity between the variant profiles used in the simulations. **A** Simulated time series of B.1.617.2 (Delta) overtaking B.1.1.7 (Alpha). **B** Simulated time series of five highly similar Omicron subvariants. **C** Same

as (A), but with two additional related variants included in the deconvolution (overspecified model). **D** Same as (B), but with one additional related variant included in the deconvolution (overspecified model). **E** Same as (B), but omitting XBB from the deconvolution (underspecified model). **F** Simulated mixture of five artificial highly related variants generated from all subsets of size four out of five informative sites.

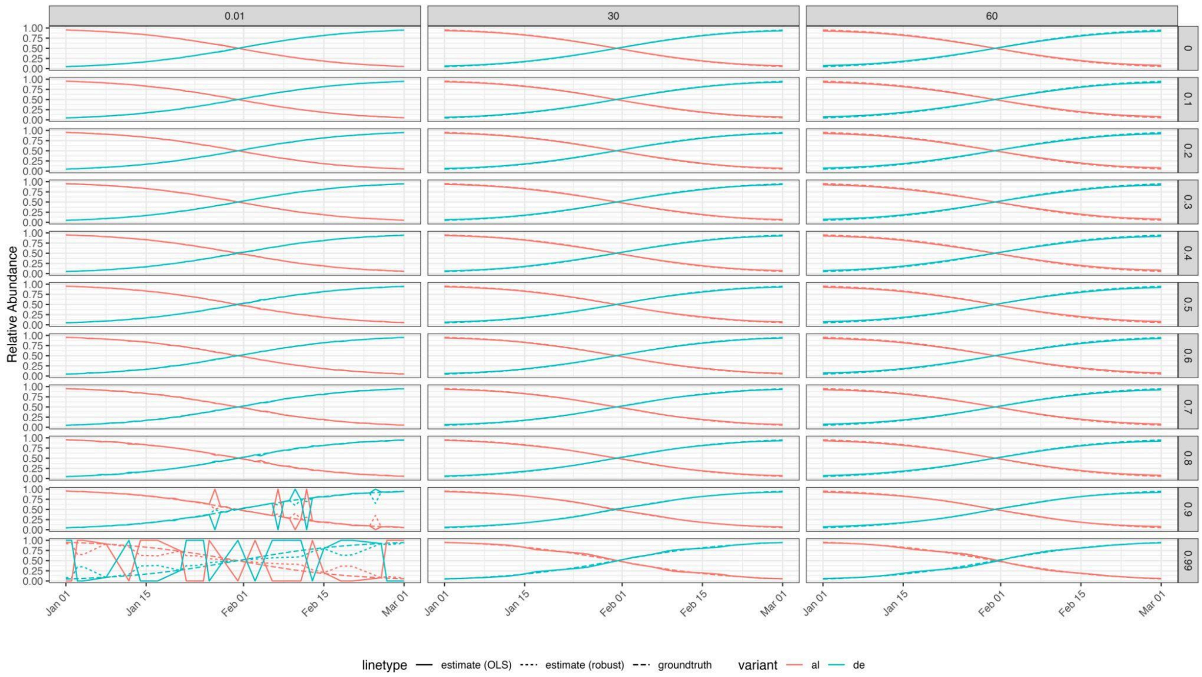

Fig E: Simulation experiments, displaying the effect of kernel smoothing on varying levels of missing value on a 60-timesteps timeseries of B.1.617.2 (de) taking over B.1.1.7. (al). Columns are different values of the smoothing bandwidth, rows are different levels of missing values. Dashed lines represent ground truth, solid lines and dotted lines show deconvolution results with the LS and SL1 loss functions, respectively.

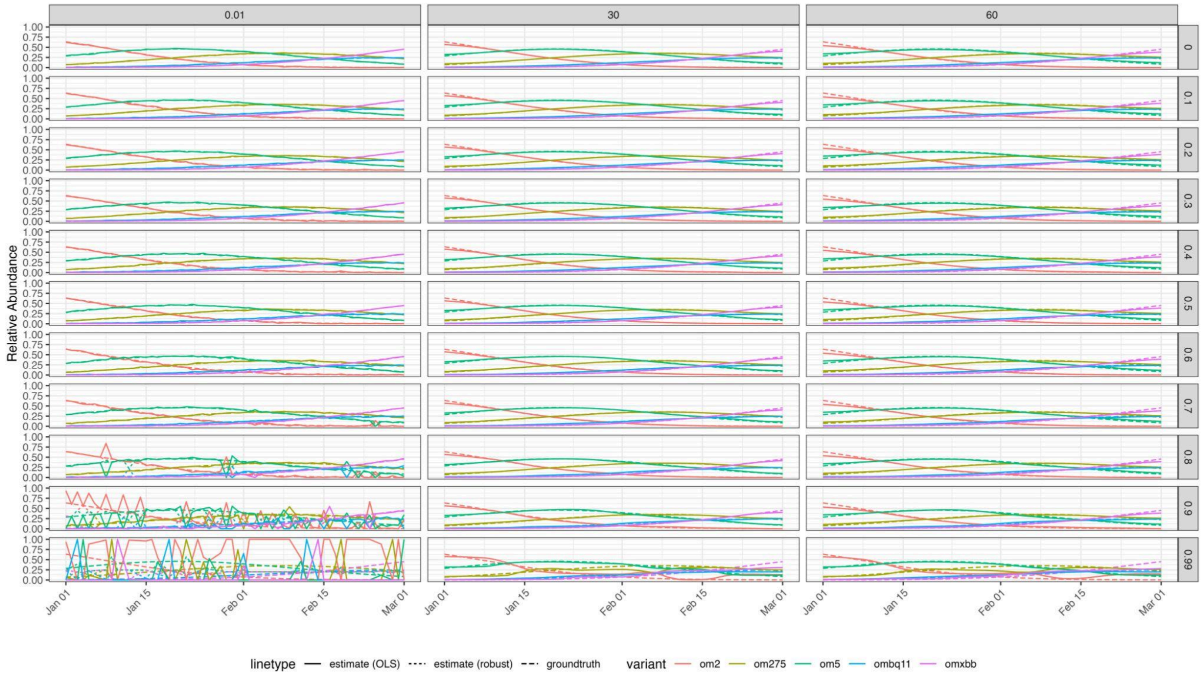

Fig F: Simulation experiments, displaying the effect of kernel smoothing on varying levels of missing value on a 60 timesteps time series of the five closely related Omicron derivatives BA.2 (om2),

BA.2.75 (om275), BA.5 (om5), BQ.1.1 (ombq11) and the recombinant XBB (omxbb). Columns are different values of the smoothing bandwidth, rows are different levels of missing values. Dashed lines represent ground truth, solid lines and dotted lines show deconvolution results with the LS and SL1 loss functions, respectively.

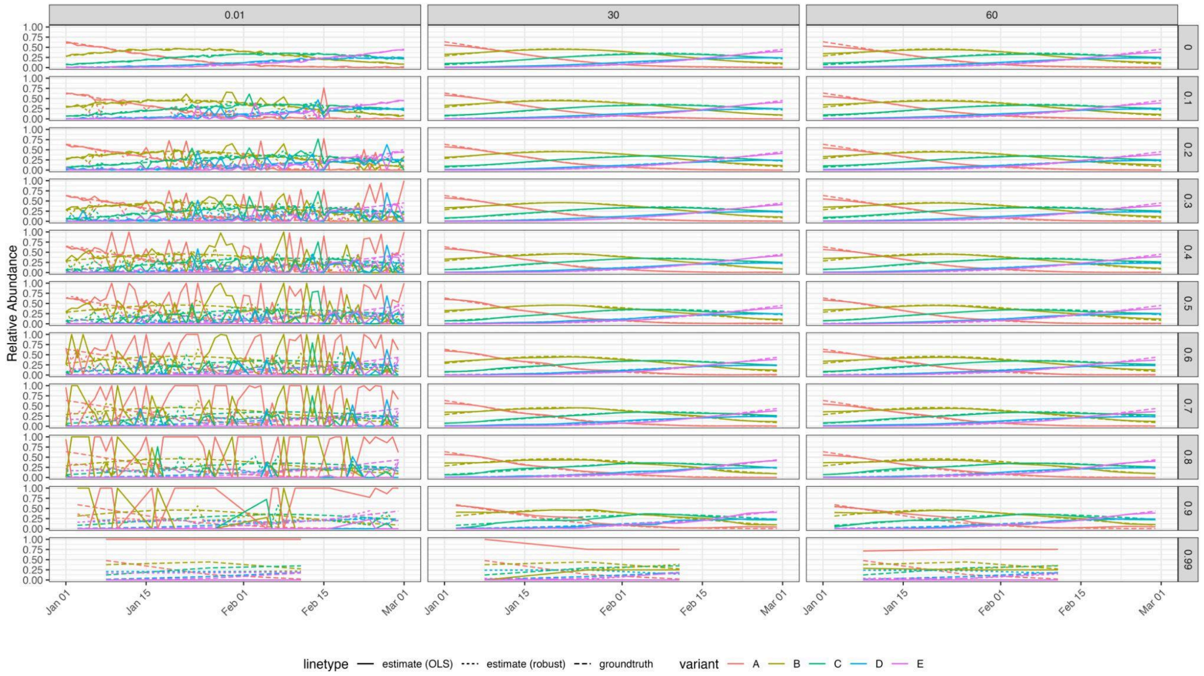

Fig G: Simulation experiments, displaying the effect of kernel smoothing on varying levels of missing value on a 60 timesteps time series of the five artificially closely related variants. Columns are different values of the smoothing bandwidth, rows are different levels of missing values. Dashed lines represent ground truth, solid lines and dotted lines show deconvolution results with the LS and SL1 loss functions, respectively.

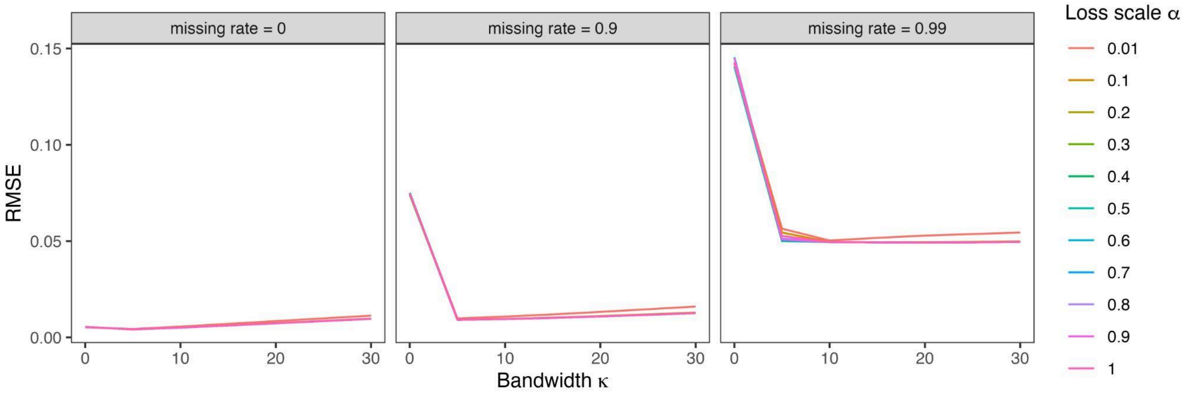

Fig H: Root Mean Square Error (RMSE) of the deconvolved simulated data, as a function of the kernel bandwidth κ and α scale parameters of the robust regression. The accuracy of the deconvolution was measured on the simulated data for 5 closely related omicron subvariants shown in Fig F in S1.

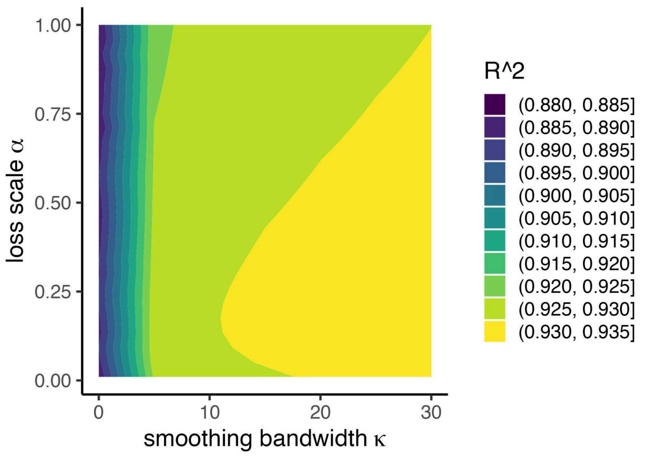

Fig I: Goodness of fit of the robust kernel deconvolution of the wastewater NGS, as a function of the kernel bandwidth κ and α scale parameters of the robust regression. Goodness of fit is evaluated by regressing on estimates of relative abundances of variants obtained from clinical sequencing data.
